# Associations among oxytocin receptor gene (*OXTR*) DNA methylation in adulthood, exposure to early life adversity, and childhood trajectories of anxiousness

**DOI:** 10.1038/s41598-017-07950-x

**Published:** 2017-08-07

**Authors:** J. P. Gouin, Q. Q. Zhou, L. Booij, M. Boivin, S. M. Côté, M. Hébert, I. Ouellet-Morin, M. Szyf, R. E. Tremblay, G. Turecki, F. Vitaro

**Affiliations:** 1 Department of Psychology, Concordia University, Montreal, Canada; 2 Sainte-Justine Hospital Research Center, University of Montreal, Montreal, Canada; 3 Research Unit on Children’s Psychosocial Maladjustment (GRIP), Laval University, Québec, Canada; 4 Institute of Genetic, Neurobiological, and Social Foundations of Child Development, Tomsk State University, Tomsk, Russian Federation; 5 Department of Social and Preventive Medicine, University of Montreal, Montreal, Canada; 6 Research Unit on Children’s Psychosocial Maladjustment (GRIP), University of Montreal, Montreal, Canada; 7 Bordeaux Population Health Research Center, INSERM and Bordeaux University, Bordeaux, France; 8 Department of Sexology, Université du Québec à Montréal, Montreal, Canada; 9 Department of Criminology, University of Montreal, Montreal, Canada; 10 Department of Pharmacology & Therapeutics, McGill University, Montreal, Canada; 11 Departments of Pediatrics and Psychology, University of Montreal, Montreal, Canada; 12 School of Public Health, University College Dublin, Dublin, Ireland; 13 Department of Psychiatry, McGill University, Montreal, Canada; 14 School of Psychoeducation, University of Montreal, Montreal, Canada; 15 School of Psychology, Laval University, Québec, Canada

## Abstract

Recent models propose deoxyribonucleic acid methylation of key neuro-regulatory genes as a molecular mechanism underlying the increased risk of mental disorder associated with early life adversity (ELA). The goal of this study was to examine the association of ELA with oxytocin receptor gene (*OXTR)* methylation among young adults. Drawing from a 21-year longitudinal cohort, we compared adulthood *OXTR* methylation frequency of 46 adults (23 males and 23 females) selected for high or low ELA exposure based on childhood socioeconomic status and exposure to physical and sexual abuse during childhood and adolescence. Associations between *OXTR* methylation and teacher-rated childhood trajectories of anxiousness were also assessed. ELA exposure was associated with one significant CpG site in the first intron among females, but not among males. Similarly, childhood trajectories of anxiousness were related to one significant CpG site within the promoter region among females, but not among males. This study suggests that females might be more sensitive to the impact of ELA on *OXTR* methylation than males.

## Introduction

Exposure to early life adversity (ELA), including physical and sexual abuse and neglect as well as poor socioeconomic conditions during childhood, are associated with increased risk for anxiety and depression later in life^1, 2^. A growing body of evidence suggests a biological embedding of early experiences leading to lasting consequences on autonomic, neuroendocrine, immune, and neural function^3^. Recent models propose deoxyribonucleic acid (DNA) methylation of key neuro-regulatory genes as an underlying molecular mechanism responsible for the increased risk for mental disorders associated with ELA exposure^4, 5^.

Gene methylation is an epigenetic process impacting transcriptional activity of DNA without altering the actual DNA nucleotide sequence. Gene methylation represents a covalent modification of DNA by the addition of a methyl group onto cytosine rings found within 5′-Cytosine-phosphate-Guanine-3′ (CpG) dinucleotide pairs^6^. Gene methylation typically influences phenotypic variation by suppressing gene expression via the prevention of transcription factors binding to their cis-acting elements in gene promoters and enhancers, and through the recruitment of methylated-DNA binding proteins that lead to the formation of a closed chromatin configuration blocking access to the DNA^6^. DNA methylation is the closest epigenetic mark to the gene itself and it is assumed to be the most stable epigenetic modification^7^.

Early life is a period particularly sensitive to the regulatory effects of epigenetic mechanisms^8^. A seminal study demonstrated that maternal care influenced DNA methylation of the glucocorticoid receptor (*GR*) in rats^9^. Offspring of low licking and grooming mothers had greater methylation of *GR* exon 17 promoter region, compared to offspring of mothers providing more maternal care. Furthermore, these changes in DNA methylation were associated with *GR* expression in the hippocampus and physiological stress responses in adulthood^9^. Other animal studies have replicated the effects of ELA exposure on DNA methylation of other candidate genes. Notably, manipulation of ELA impacted methylation of the *ESR1, CRH, BDNF*, and *AVP* genes, providing strong evidence for the impact of ELA on gene methylation^9–13^.

Early life adversity also leads to genome-wide methylation modifications. Among rhesus macaques, differential rearing conditions (maternal vs. surrogate peer rearing) led to global methylation differences in as many as ~1300 distinct gene promoters in T-cells and in the prefrontal cortex^14^. In humans, institutionalized children exhibited differential methylation in 800 gene promoters from whole blood, compared to children raised by their biological parents^15^. Furthermore, genome-wide promoter methylation profiling revealed that ELA was associated with a distinct epigenetic signature in peripheral blood leukocytes and hippocampal tissues in adulthood^16–19^. Notably, the epigenetic profile was more strongly associated with early life adversity than adversity later in life^17, 20, 21^. This suggests that ELA exposure leads to genome-wide epigenetic changes in peripheral and central tissues.

ELA has been associated with DNA methylation of candidate genes involved in key neuro-regulatory functions among both healthy individuals and individuals suffering from mental disorders. Indeed, childhood adversity has been associated with greater methylation of different candidate genes, such as *NR3C1, FKBP5, SLC6A4*, and *BDNF*, in peripheral blood leukocytes of healthy and depressed children and adults^22–30^. Furthermore, post-mortem analysis of suicide victims’ hippocampal tissues revealed that a history of childhood abuse was related to greater methylation of *NR3C1* as well as the ribosomal ribonucleic acid (RNA) genes^19, 31^, indicating that DNA methylation changes were also observed in central tissues. Importantly, DNA methylation mediated the impact of early adversity on later health outcomes^32–34^.

The oxytocinergic system is a key neurobiological system involved in social behaviors. In animal studies, pharmacological oxytocin (OT) manipulation facilitated social recognition, social bonding, maternal care, aggression, and sexual activity^35^. In humans, intra-nasal oxytocin administration modulated social cognition and behaviors^36^. The oxytocinergic system is also associated with anxiety and depression. In animal models, oxytocin receptor gene (*OXTR*) knockout mice did not benefit from the antidepressant effect of mating behavior, compared to wild type mice^37, 38^. Further, an oxytocin receptor agonist had antidepressant properties^39, 40^, while an oxytocin receptor antagonist blocked the buffering effect of social relationships on depressive behavior^41, 42^. In humans, depression and anxiety have been associated with dysregulated plasma OT^43–47^. Further, *OXTR* polymorphisms have been associated with stress reactivity and psychological distress^48–52^, especially in the context of ELA exposure^53–56^. Importantly, oxytocin appears to have a sexually dimorphic role, with females showing a greater association between oxytocin and prosocial behaviors than males^57^.

The oxytocinergic system shows considerable plasticity in response to early life events. In rodents and primate studies, ELA exposure was associated with changes in oxytocin receptor binding and expression in limbic and prefrontal areas, as well as altered cerebrospinal fluid OT levels^58–68^. In humans, individuals with a history of ELA had lower cerebrospinal fluid OT levels^69^ and exhibited altered cortisol, limbic, and caregiving responses to intra-nasal OT administration^70–72^, compared to individuals without an ELA history. Moreover, children raised in an orphanage had lower OT production during mother-infant interactions, compared to children raised by their biological parents^73^.

To date, only a few studies have investigated methylation of oxytocinergic genes. A single oxytocin receptor gene (*OXTR*) localized on chromosome 3 at locus 3p25 has been identified^74^. Early epigenetic studies using luciferase reporter gene assays showed that methylation of a CpG island within the first intron influenced transcriptional activity^75^. In a seminal paper, five CpG dinucleotides of this CpG island had significantly higher DNA methylation in patients with autism, compared to healthy control subjects^76^. This difference was found not only in blood DNA, but also in DNA extracted from brain tissue^76^, providing some evidence for correspondence of brain and blood DNA methylation for this gene. Subsequent studies examining this same CpG island found that differences in methylation frequency from whole blood were related to individual differences in anorexia nervosa symptoms, child conduct problems, unemotional and callous-unemotional trait, acute psychosocial stress, perception of ambiguous social stimuli, and limbic activation in response to fear^77–82^. Furthermore, studies investigating other CpG sites within *OXTR* have found significant associations with social anxiety and depressive disorders^83–85^, as well as pessimism and interpersonal distrust^86^.

A few studies have investigated the association of ELA with *OXTR* methylation in adulthood. In a rodent study, natural variations in maternal care were associated with altered *OXTR* DNA methylation in central and peripheral tissues among adult rats^87^. In humans, an epidemiological study reported that low childhood socioeconomic status (SES) was associated with increased *OXTR* DNA methylation in non-promoter regions of the gene, but was not related to methylation of CpG sites within the promoter region^88^. Moreover, poorer maternal care in childhood was associated with greater *OXTR* methylation within exon 3 in peripheral blood cells^89^. Furthermore, in a sample of African-Americans with low socio-economic status, early child abuse was associated with higher *OXTR* DNA methylation of two CpG sites in exon 3 in whole blood, but not at the promoter region^90^. These data provide preliminary evidence that ELA is associated with methylation of different regions within *OXTR*.

Although past studies have examined the association of ELA with *OXTR* methylation in adulthood, they have focused on a limited subset of regulatory regions within *OXTR*. Notably, past studies have focused on a limited number of CpG sites within the promoter region. Promoter regions are interesting targets because they are enriched in both CpG dinucleotides and binding sites for transcriptional activators and repressors. Methylation of such regions can hinder the proper interaction between DNA and the transcription factors. Disruptions of such interactions bring about inhibition of downstream gene expression^91^. Furthermore, chromatin immune-precipitation (ChIP)-sequencing indicates that there are two enhancer elements within the 3^rd^ intron, which might be subjected to regulation by DNA methylation^92^. Enhancer elements are short distal regions involved in the co-regulation of gene transcription, usually acting cis to the promoter elements^93^. Moreover, past studies have used retrospective and limited assessments of ELA.

The primary aim of this study was to examine the predictive association between prospectively assessed ELA and later peripheral *OXTR* methylation in adulthood. Various regulatory regions of the gene were assessed in order to identify key CpG sites sensitive to ELA. Given that methylation mediated the impact of ELA on later health outcomes in animal and human studies^9, 32, 33^, a secondary aim of this study was to evaluate the associations between *OXTR* methylation and childhood trajectories of anxiousness and disruptiveness. Furthermore, given the sexually dimorphic role of oxytocin in social behaviors^57^, sex-specific analyses were also conducted. We hypothesized that ELA exposure would be associated with greater *OXTR* methylation, that *OXTR* methylation would be related to childhood trajectories of anxiousness and disruptiveness, and that females would show a stronger association between ELA and *OXTR* methylation.

## Methods

### Participants

Participants were recruited from l’Étude longitudinale des enfants de maternelle au Québec (ÉLEMQ), a longitudinal study of 3785 children initially recruited while they were attending kindergarten in francophone schools in Québec. From this larger sample, a randomly selected, representative group of 2000 boys and girls was followed longitudinally. The cohort was followed yearly from ages 6–12, and then in mid-adolescence (mean age = 15), in emerging adulthood (mean age = 21) and in adulthood (mean age = 27). At age 27, participants provided a blood sample for epigenetic analysis. A subset of these participants was selected for exposure to high or low levels of early adversity.

In order to create two groups with differential ELA exposure, 46 participants with available DNA were selected based on their scores on both childhood SES and early exposure to abuse (see below). These two aspects were considered given that childhood socioeconomic status has a different methylation signature than childhood abuse^17, 94^, suggesting that these two factors might independently predict adulthood methylation profiles. Specifically, two extreme groups were established on the basis of children’s z-scores for both indices calculated on the entire cohort. Participants in the high ELA group (n = 24) scored low on the SES index, but high on the Abuse index, whereas participants in the low ELA group (n = 22) scored high on the SES index, but low on the Abuse index. An equal number of males and females were selected in each group. To reduce genetic admixture, only Caucasian individuals of Western European Ancestry were included in the study. This study was approved by the Hospital Ste-Justine Research Ethics Board. The methods were carried out in accordance with the relevant guidelines and regulations. All participants provided informed consent.

### Psychosocial Measures

#### Childhood Socio-Economic Status (SES)

Childhood socioeconomic status was well characterized in this cohort using five relevant socioeconomic indicators collected prospectively across childhood. Maternal and paternal years of schooling, mean maternal and paternal occupational prestige, and mean family income were assessed at 8 occasions when the participants were aged 6–12, and were then averaged. Confirmatory factor analysis aggregating the 5 indicators was used to create a general childhood SES score for each individual.

#### Childhood Abuse Index

Exposure to physical and sexual abuse during childhood and adolescence was assessed retrospectively at age 21^95^. Eight items of the Parent–Child Conflict Tactics Scale^96^ were used to evaluate child physical abuse by a mother or father figure. Participants indicated how often they experienced each of the following items during their childhood: severe physical abuse (4 items; e.g., hit you with an object, brutally threw you against a wall) and very severe physical abuse (4 items; e.g., threatened you with a weapon, beat you up over and over). Five questions regarding childhood sexual abuse were adapted from the Adverse Childhood Experiences Questionnaire^97^ and from the Sexually Victimized Children Questionnaire^98^. Participants were asked if they had experienced any unwanted sexual acts against their will before the age of 18 years, including exhibitionism (being forced to look or forced to show genitals), sexual fondling or touching, and completed or attempted sexual intercourse by use of bribes or threats, force, or drugs and/or alcohol. The scores from these scales were combined using confirmatory factor analysis to form an overall Abuse index.

#### Childhood trajectories of anxiousness and disruptiveness

Items from the teacher-rated Social Behavior Questionnaire^99^, administered yearly from age 6 to 12, were considered to evaluate trajectories of childhood anxiousness and childhood disruptiveness. The trajectories of childhood anxiousness were assessed using the following items: fearful or afraid of things or new situations; is worried, worries about many things; cries easily; has a tendency to work alone; looks sad, unhappy, tearful; easily distracted (age 6 Cronbach’s α = 0.74). The trajectories of childhood disruptiveness items included: destroys one’s own things or those of others; fights with other kids; is not liked by peers; irritable; disobedient; lies; mistreats, intimidates peers; does not share material used for a particular task; blames others; inconsiderate of others; hits and kicks others; fidgets, squirms, cannot keep still; agitated, always running and jumping, restless (age 6 Cronbach’s α = 0.90). The trajectories (low, average and high) were characterized using semi-parametric group-based modeling^100^. Children classified in the *low* trajectory were consistently rated as displaying low levels of anxiousness or disruptiveness, while participants in the *high* trajectory had elevated ratings of anxiousness or disruptiveness throughout childhood. Participants in the *average* trajectories varied between high and low yearly ratings of anxiousness or disruptiveness from age 6 to 12.

### OXTR Target Sequence Selection

**Figure 1:**
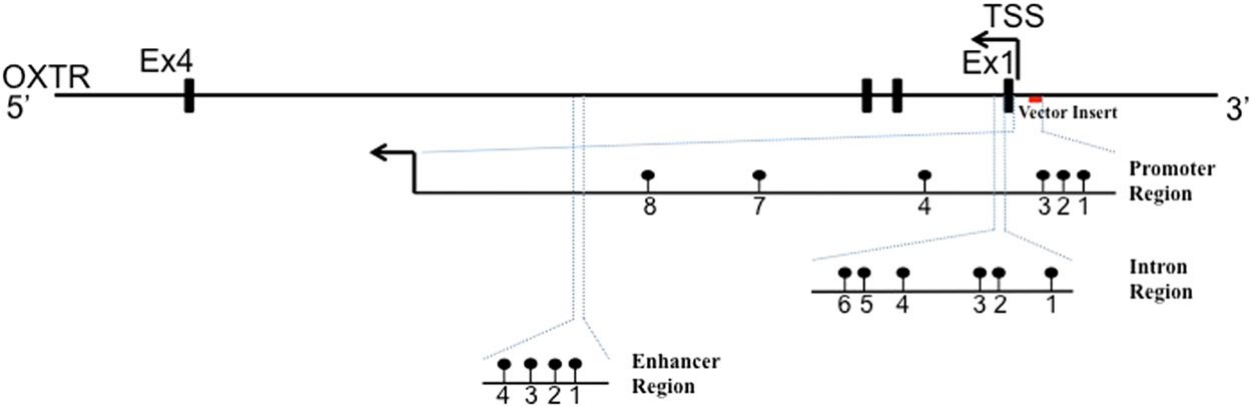
Schematic representation of the genomic regions of interest within the *OXTR* gene. The promoter region (chr3: 8811303-8811915) was identified using the H3K4Me3 signals from the ENCODE database. The Intron 1 region (chr3: 8810699-8810875) was identified based on past literature. The Enhancer region (chr3: 8806851-8806950) was identified using the H3K4Me1 signal from the ENCODE database. The segment in red represents the genomic location of luciferase reporter construct tested. The genomic coordinates of the specific CpGs tested are found in the supplementary information.

To identify potential regulatory regions of the gene, ChIP-sequencing experiment data available in an open-access database part of the ENCODE histone project were used^101^. H3K4Me1 and H3K4Me3 signal tracks using PBMC and HepG2 cell lines were obtained through the ENCODE database and visualized via the UCSC genome browser using the genome build hg19^102^. H3K4Me1 and H3K4Me3 are specific histone modifying proteins that are indicative of active enhancers and promoters respectively. Regions of peak signal intensity are regions within the DNA sequence that bind specifically to H3K4Me1 and H3K4Me3 antibodies with high signal strength, representing robust areas of protein binding when taking into account the background noise inherent in the ChIP-sequencing procedure^103^. The exact DNA sequence corresponding to a signal region was extracted with full annotation of its CpG dinucleotides. For the promoter, the region length was defined from 700 base pairs upstream of the gene to the transcription start site (TSS). For enhancers, a DNA sequence was extracted 100 base pairs upstream and downstream from the H3K4Me1 signal peak, which corresponds to the region with the highest confidence of protein binding activity^101^. As depicted in Fig. 1, CpG dinucleotides within 3 genomic regions were selected for the present study.

### Sample Preparation

Peripheral blood samples were collected from participants and stored in EDTA coated tubes at 4 °C before extraction. DNA extraction was performed using the QIAamp DNA Mini Kit (Qiagen, #51304) according to the manufacturer’s instructions and stored at −20 °C. Extracted DNA was stored in a −80 °C freezer.

### Pyrosequencing

To investigate *OXTR* DNA methylation, a total of three sets of outside primers and four sets of inside primers were developed to probe all CpG sites within the target regions (promoter, intron 1, enhancers). The nested reverse primers were biotinylated for pyrosequencing (IDT Technologies). 500 ng of DNA was treated with sodium bisulphite (EZ Methylation Gold, Zymo Research) and underwent two rounds of PCR amplification (#1, 95 C × 15 min, [94 × 1 min, Primer TM * 1 min, 72 C * 1 min] for 35 cycles, 72 C * 10 min; #2 95 C × 15 min, [94 × 1 min, Primer TM * 1 min, 72 C * 1 min] for 40 cycles, 72 C * 10 min). The subsequent PCR product then underwent gel electrophoresis to confirm the purity and the success of the amplification protocol. 20 ul of the PCR product was then used to perform pyrosequencing using PyroMarkQ24 (Qiagen) according to the manufacturer protocol. All PCR primers used are listed in Supplementary Table S1. The methylation percentage at each individual CpG site was analyzed and exported using PyroMark Q24 software (Qiagen). Triplicate analyses were performed per sample to assure accuracy. Data are reported as the average of the triplicates. We were unable to quantify Enhancer 2 methylation because of difficulties in designing the sequencing primer that adheres to the target segment with high affinity due to the repetitive composition of the Enhancer Region 2 DNA sequence. A list of all successfully quantified CpG sites with their respective genomic positions is listed in Supplementary Table S2. Site-specific methylation analyses were performed at CFI Imaging and Molecular Biology Platform at McGill University in the Department of Pharmacology and Therapeutics.

### Luciferase Reporter Construct

Three artificial constructs of the *OXTR* promoter were generated (602 base pair whole promoter sense, 602 base pair whole promoter antisense, and 50 base pair promoter) using primers with artificially introduced restriction sites to allow for sense and antisense insertion of DNA fragments. A sense sequence is a DNA sequence in its 5′ to 3′ direction while an antisense sequence is the complement of the sense sequence in the reverse 3′ to 5′ direction. Human genomic DNA from whole blood was subject to two rounds of PCR amplification with HotStar Taq DNA Polymerase (Qiagen). The resulting DNA was digested with BamHI and HindIII and subcloned into the CpG-free pCpGL-basic luciferase reporter plasmid. SssI DNA methyltransferase (New England Biolabs) was then used to methylate the plasmid construct *in vitro*.

The pCpGL-basic plasmid is a construct with no-inherent CpG sites; methylation of such plasmid occurs exclusively on the introduced gene vector sequence. This strategy ensures that there is no confounding effect of methylation sites along the whole plasmid construct. The resultant constructs were then validated via sequencing (Genome Quebec, Montreal). Both the methylated and non-methylated plasmids were then transfected into HEK2 93 (human embryonic kidney) cells using standard methods. HEK293 cells were cultured in DMEM 1X (GIBCO, Invitrogen) with 10% fetal bovine serum (GIBCO, Invitrogen), plated and transfected using a standard calcium phosphate method^104^. Cells were lysed and harvested 48 hours post transfection, and luciferase activity was measured via the Luciferase Assay System (Promega). The primers used are listed in Supplementary Table S3.

### Statistical Analysis

All data were inspected for normality of the distributions. All variables approximated the normal distribution except for promoter CpG3. The distribution of promoter CpG 3 showed a significant departure from normality driven by one outlier value. However, even when this outlier value was removed from the analysis, the effect of ELA remained statistically significant after a Bonferroni correction. Given the small sample size, we decided to retain all the data in the analyses. Multivariate and univariate general linear models explored the associations among ELA, *OXTR* methylation, and childhood trajectories of anxiousness and disruptiveness. Weighted posterior probabilities were used to test the associations between *OXTR* DNA methylation and the childhood trajectories of anxiousness and disruptiveness^100^. It was hypothesized that *OXTR* methylation would mediate the relationships between ELA and childhood trajectories of anxiousness among females. Mediation analyses were performed by testing the significance of the indirect effect using bootstrapping resampling methods with the PROCESS module for SPSS^105^. Using this approach, the indirect effect is considered statistically significant when its confidence interval does not include zero. Logistic regressions were used for the mediation given the categorical dependent variable. This method is superior to traditional tests of mediation because it does not require large sample sizes nor does it assume normal distribution of the indirect effect^106^. For the luciferase assay, paired two-tailed t-tests were used to examine the signal differences in control and test plasmids. Given the small number of missing data, mean substitution was used to deal with missing data in multivariate tests. All the data available were used for the univariate analyses. Statistical significance was reported at *p* < 0.05. Family-wise Bonferroni corrections were used to limit type I error due to multiple comparisons. Given the risk of type II error in a small sample, interpretation focused on large effect sizes (Cohen’s *d* > 0.80). Effect sizes quantified using Cohen’s *d* were calculated by dividing the mean difference between the two groups by the pooled standard deviation^107^. Partial*η* ^2^ was used as measure of effect size in multivariate analyses. It represents the proportion of the total variance in a dependent variable that is associated with the independent grouping variable after partialing out the effects of other independent variables and interaction effects^108^. Statistical analyses were conducted using SPSS v. 22.

## Results

### *OXTR* DNA Methylation and Early Life Adversity

**Figure 2:**
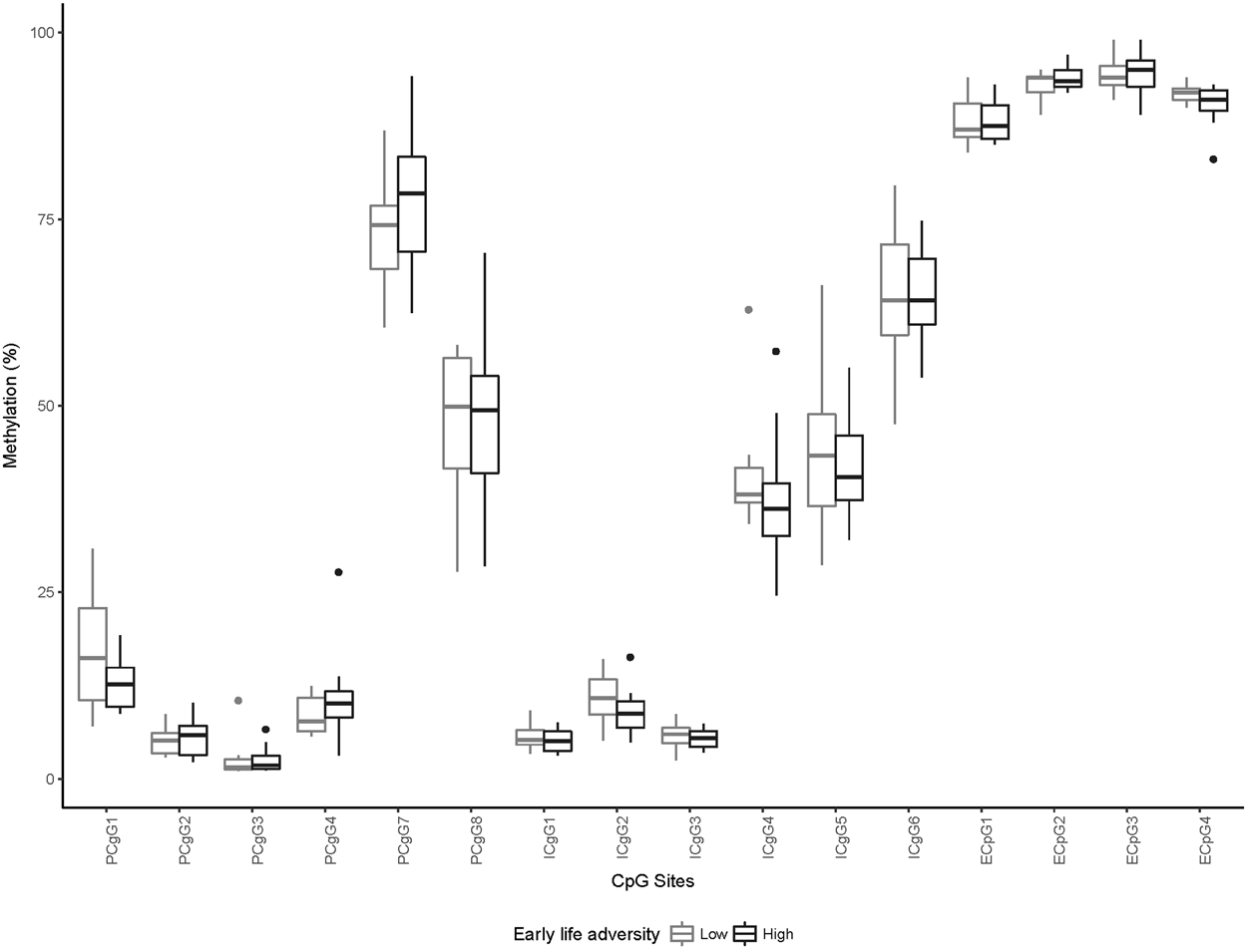
*OXTR* Methylation as a Function of ELA Exposure in Males. *Represents statistically significant ELA group differences at *p* < 0.05; **represents statistically significant difference after a Bonferonni correction where *p* < 0.003.

**Figure 3:**
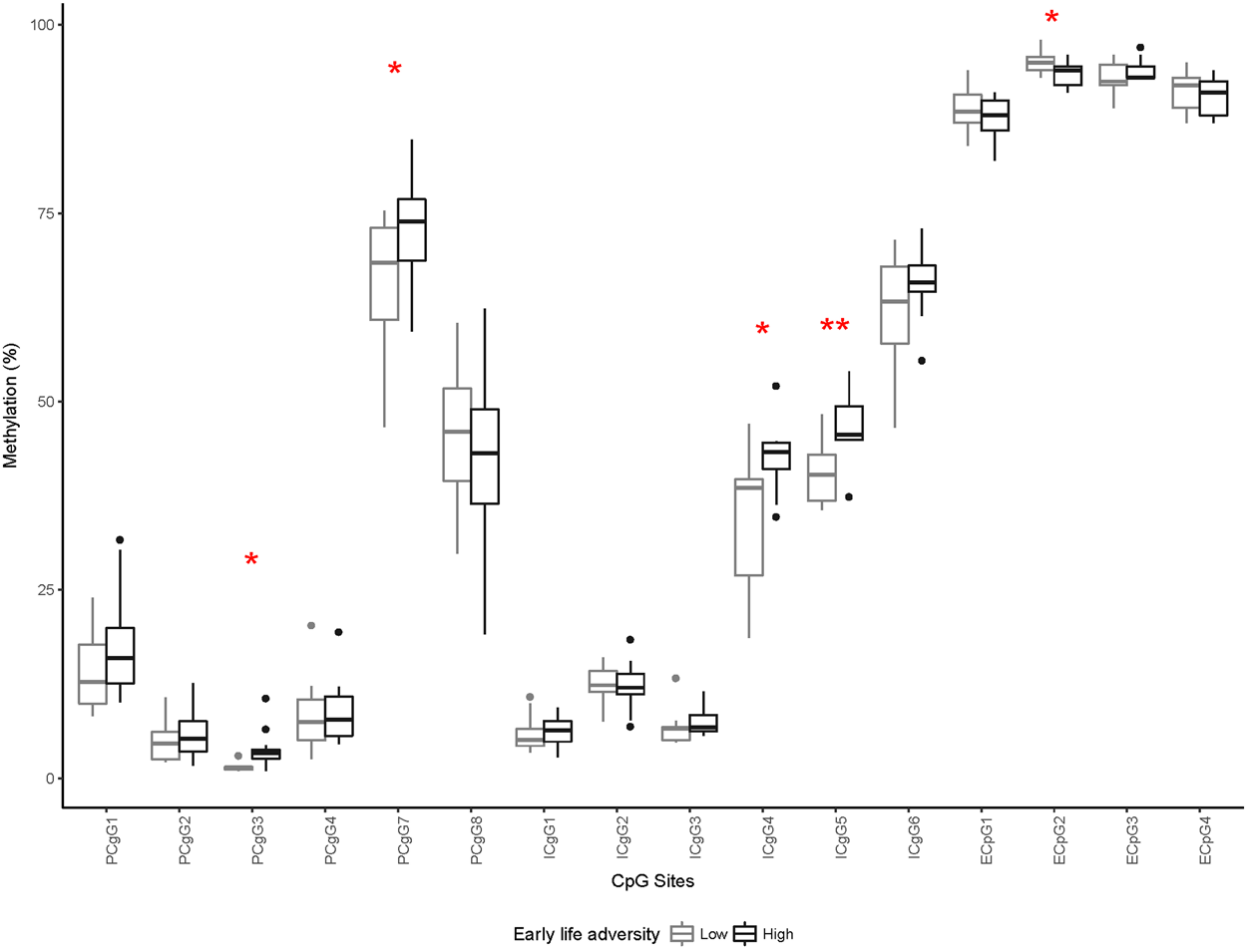
*OXTR* Methylation as a Function of ELA Exposure in Females. *Represents statistically significant ELA group differences at *p* < 0.05; **represents statistically significant difference after a Bonferonni correction where *p* < 0.003.

DNA methylation frequency of three genomic regions within *OXTR* was quantified in 46 individuals via pyrosequencing. All participants were 27 years of age and of Western European ancestry, with an equal number of males and females (23 males, 23 females) and a similar number of individuals in high (n = 24) and low (n = 22) ELA groups. DNA methylation frequency across the 16 CpG sites that were successfully quantified ranged from 1% to 95%. The average methylation frequencies of all CpG sites as a function of ELA exposure for males and females are presented in Figs 2 and 3, respectively.

There was no overall DNA methylation difference between the high and low ELA groups, *F*(16,29) = 1.03, *p* = 0.46, *η* ^2^ = 0.36. Given that the association between ELA and *OXTR* methylation varied across genomic regions^109, 110^, the associations of ELA with individual CpG sites were evaluated. Individuals in the high ELA group had significantly greater promoter CpG 7 methylation, compared to participants in the low ELA group, *F* (1,44) = 5.3, *p* = 0.03, *d* = 0.69, mean methylation difference = 5.9%. However, this difference was no longer statistically significant after a Bonferroni correction, adjusted *p* = 0.48.

### Sex Differences in *OXTR* Methylation

Given previous evidence showing a sexually dimorphic effect of OT on social behavior^111^, we examined sex differences in *OXTR* methylation, regardless of ELA. Three CpG sites exhibited significant sex differences. Females had significantly lower DNA methylation in promoter CpG 7 than males, *F*(1,44) = 6.0, *p* = 0.02, *d* = 0.64. In contrast, females had higher DNA methylation in Intron CpG 2, *F*(1,44) = 7.49, *p* = 0.01, *d* = 0.82, and Intron CpG 3, *F*(1,44) = 7.11, *p* = 0.01, *d* = 0.81, compared to males.

### ELA and *OXTR* Methylation Among Females and Males

The association between ELA and global *OXTR* methylation was a trend, *F*(6,16) = 3.06, *p* = 0.09, partial *η* ^2^ = 0.89, among females, while there was no significant association among males, *F*(6,16) = 0.62, *p* = 0.79, partial *η* ^2^ = 0.62. Tables 1 and 2 provide ELA group differences for each individual CpG site for females and males respectively. For females, the association between ELA and *OXTR* methylation had a large effect size, i.e., a Cohen’s *d* > 0.80, for 5 CpG sites. For promoter CpG 3, promoter CpG 7, intron 1 CpG 4, and intron 1 CpG 5, females in the high ELA group had higher *OXTR* DNA methylation than females in the low ELA group. In contrast, high ELA was associated with lower methylation for enhancer 1 CpG 2. After applying a Bonferroni correction for multiple comparisons, there was a significant group difference only for intron 1 CpG 5. There was no ELA group difference with a large effect size in males.

**Table 1:** Effect Sizes of the Association Between ELA and *OXTR* Methylation for the Different CpG Sites among Females.

*CpG sites*	*F*	*p-value*	Cohen’s d
Promoter CpG 1	1.53	0.22	0.52
Promoter CpG 2	0.38	0.54	0.26
Promoter CpG 3	8.18	0.009*	1.22
Promoter CpG 4	0.03	0.87	0.07
Promoter CpG 7	5.62	0.03*	1.00
Promoter CpG 8	0.28	0.60	0.22
Intron 1 CpG 1	0.10	0.75	0.14
Intron 1 CpG 2	0.05	0.82	0.10
Intron 1 CpG 3	1.03	0.32	0.42
Intron 1 CpG 4	5.98	0.02*	1.08
Intron 1 CpG 5	12.27	0.002**	1.47
Intron 1 CpG 6	1.82	0.19	0.56
Enhancer 1 CpG 1	0.73	0.40	0.38
Enhancer 1 CpG 2	4.77	0.04*	0.97
Enhancer 1 CpG 3	1.27	0.27	0.60
Enhancer 1 CpG 4	0.64	0.43	0.35

*p < 0.05, **significant after Bonferroni correction where *p* < 0.003.

**Table 2:** Effect Sizes of the Association between ELA and *OXTR* Methylation for the Different CpG Sites among Males.

*CpG Sites*	*F*	*p-value*	Cohen’s d
Promoter CpG 1	1.92	0.18	0.60
Promoter CpG 2	0.19	0.67	0.18
Promoter CpG 3	0.009	0.93	0.04
Promoter CpG 4	1.04	0.32	0.44
Promoter CpG 7	1.14	0.30	0.45
Promoter CpG 8	0.02	0.88	0.05
Intron 1 CpG 1	0.55	0.47	0.32
Intron 1 CpG 2	1.87	0.19	0.57
Intron 1 CpG 3	0.24	0.63	0.21
Intron 1 CpG 4	1.05	0.32	0.46
Intron 1 CpG 5	0.26	0.62	0.21
Intron 1 CpG 6	0.009	0.93	0.04
Enhancer 1 CpG 1	0.05	0.83	0.08
Enhancer 1 CpG 2	1.61	0.22	0.53
Enhancer 1 CpG 3	0.06	0.80	0.11
Enhancer 1 CpG 4	2.73	0.11	0.70

*p < 0.05, **significant after Bonferroni correction where *p* < 0.003.

### *OXTR* Methylation and Childhood Trajectories of Anxiousness and Disruptiveness

In this sample selected for high and low ELA exposure, 39.13% of the participants belonged to the low childhood anxiousness trajectory, 47.83% were assigned to average childhood anxiousness, and 13.04% displayed elevated anxiousness throughout childhood. There was an equivalent number of males and females in each trajectory, *χ*2 (45) = 0.0001, *p* = 0.99. For the childhood disruptiveness trajectory, 52.17% of the participants were assigned to the low trajectory, 28.26% to the average trajectory, and 19.56% to the high trajectory. There was no significant sex difference in the childhood disruptiveness trajectories, *χ*2(45) = 3.19, *p* = 0.20.

**Figure 4:**
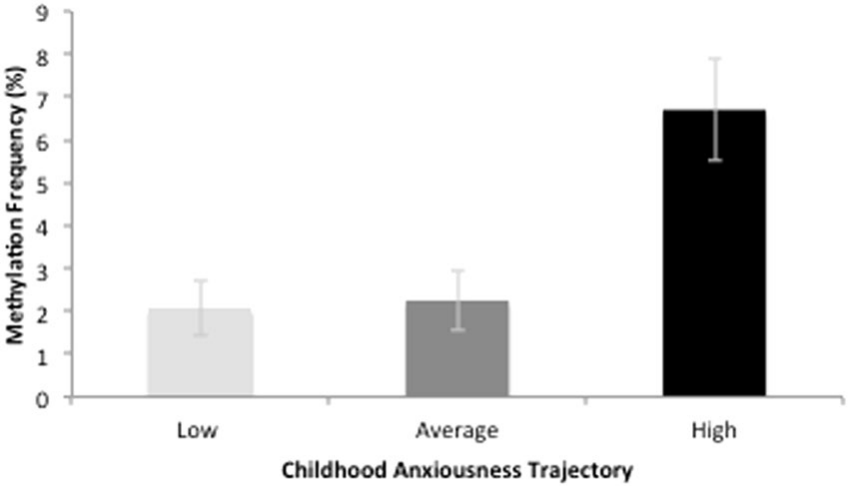
Associations between *OXTR* methylation and childhood trajectories of anxiousness. Figure 4 depicts the association between promoter CpG 7 methylation and childhood anxiousness. Individuals within the high childhood anxiousness trajectory had significant greater methylation, compared to participants in the low and average trajectories. Error bars represent standard error of the means.

A MANOVA tested the associations between childhood trajectories of anxiousness and methylation of the 5 *OXTR* CpG sites that showed large effect sizes with ELA. *OXTR* methylation was significantly related to childhood anxiousness among females, *F*(5,17) = 4.03, *p* = 0.01, partial *η* ^2^ = 0.54, but not among males, *F*(5,17) = 1.10, *p* = 0.40, partial *η* ^2^ = 0.37. Post-hoc tests indicated that for promoter CpG 3, *F*(2,20) = 4.45, *p* = 0.03, partial *η* ^2^ = 0.31, and promoter CpG 7, *F*(2,30) = 8.89, *p* = 0.002, partial *η* ^2^ = 0.47, higher methylation was associated with greater childhood anxiousness. However, only the association of promoter CpG 7 with childhood anxiousness survived correction for multiple comparisons. Intron 1 CpG 4, Intron1 CpG 5, and Enhancer 1 CpG 2 were not significantly related to childhood anxiousness among females, all *p*-values > 0.07. Moreover, for both females, *F*(5,17) = 1.84, *p* = 0.16, partial *η* ^2^ = 0.35, and males, *F*(5,17) = 0.57, *p* = 0.11, partial *η* ^2^ = 0.14, there was no significant association between childhood trajectory of disruptiveness and *OXTR* methylation. Figure 4 displays the relationship between *OXTR* methylation and childhood trajectories of anxiousness.

### Exploratory mediation analysis

**Figure 5:**
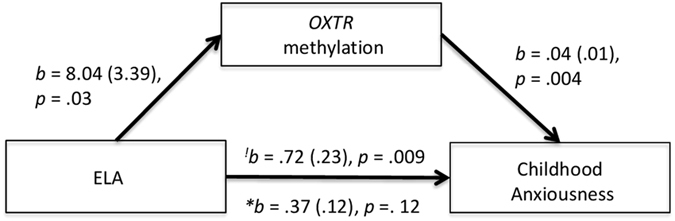
Mediation model. ^!^Indicates the direct effect of ELA on childhood anxiousness (*c path*); *indicates the direct effect path coefficient after adjusting for all the other effects in the model (*c’ path*). In this model, the confidence interval of the indirect effect does not include zero, indicating that the effect is statistically different from zero.

Prior analyses indicated that there were large Cohen’s d effect sizes for the associations among ELA, *OXTR* DNA methylation at the promoter CpG 7 site, and childhood trajectories of anxiousness. We conducted an exploratory analysis to examine whether *OXTR* methylation may act as a mediator of the association between ELA and childhood trajectories of anxiousness. Given the sex-specific associations observed, the mediation models tested whether *OXTR* methylation mediated the relationship between ELA and childhood trajectories of anxiousness in the female subsample only. The indirect effect tested the extent to which the association between ELA and childhood anxiousness is explained by their common association with *OXTR* methylation. The indirect effect for promoter CpG 7 was statistically significant, *b* = 0.35 (SE = 0.17), CI (0.07–0.78). In contrast, the indirect effect of an opposite model whereby promoter CpG 7 methylation predicted early life adversity through increased anxiousness was not statistically significant, b = 0.75, SE = 1.23 CI: −0.03–5.85. However, these results should be interpreted as exploratory given that not all individual paths from the model remained significant after correction for multiple comparisons. The mediation model is illustrated in Fig. 5.

### Functional validation of the differentially methylated region in *OXTR*

**Figure 6:**
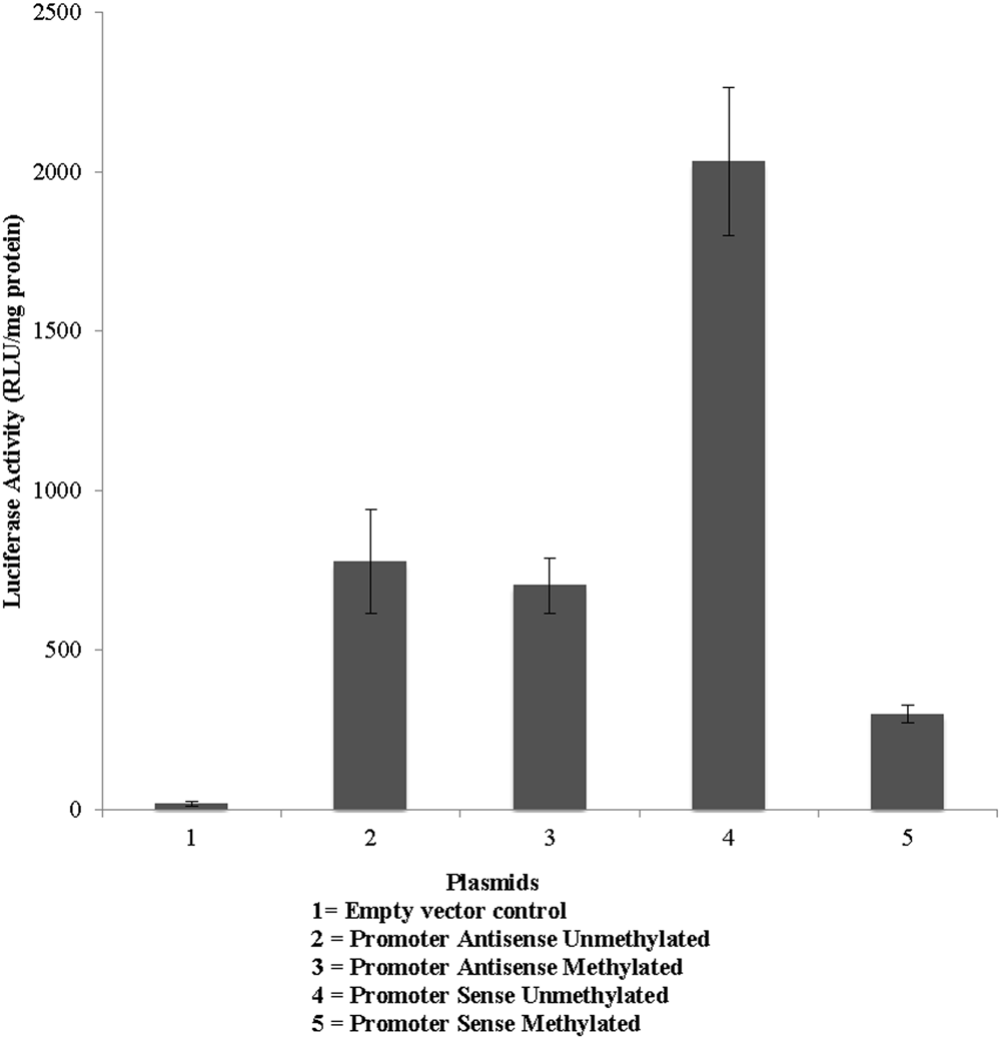
*In vitro* methylation regulates gene expression at *OXTR* promoter. Normalized luciferase activity (RLU/mg protein) in the HEK293 cell line for the antisense, methylated antisense, sense, and methylated sense promoter constructs are shown. Values are expressed as means $\pm$ standard error of the mean.

While there were significant correlations among ELA, *OXTR* DNA methylation within the promoter, and childhood trajectories of anxiousness, it is still unclear whether methylation of CpG sites within the promoter does indeed have an effect on the transcriptional machinery that may alter *OXTR* expression or other affected downstream genes. To determine the functional activity of the CpG within the promoter, we introduced the differentially methylated regions in *OXTR* (Ctrl-no insert, promoter sense, promoter antisense, and promoter subregion) to the pCpGL-basic CpG-free luciferase reporter plasmid and performed *in vitro* methylation with SSSI methyltransferase. Since the plasmid and the reporter do not contain CpG sequences, SSSI methylates the *OXTR* regions exclusively. We could therefore measure the effects of DNA methylation of this *OXTR* region without confounding effects of vector methylation. Introduction of the putative promoter region to the reporter vector induced reporter luciferase activity, confirming that the region is indeed an active promoter. We then compared the luciferase activity driven by the unmethylated promoter with the unmethylated promoter sense plasmid construct, the empty vector and methylated and unmethylated anti-sense constructs (*Promoter sense unmethylated* versus *sense methylated*, *t* = 7.38, *p* = 0.002. *promoter sense unmethylated* versus *antisense unmethylated*, *t* = −4.41, *p* = 0.01, *promoter sense methylated* versus *antisense unmethylated*, *t* = 2.81, *p* = 0.04, *promoter antisense unmethylated* versus *promoter antisense methylated, t* = 0.*40, p* = 0.*70*) (see Fig. 6). These data are consistent with the idea that CpG sites within the promoter region are important for regulating the expression of the *OXTR* gene.

## Discussion

We investigated associations among ELA, *OXTR* DNA methylation, and childhood trajectories of anxiousness and disruptiveness among young adults. Among females, one CpG site within the first intron was significantly associated with ELA and one CpG site within the promoter was significantly related to childhood trajectories of anxiousness. No significant associations were found between ELA, childhood anxiousness or disruptiveness, and OXTR methylation among males. Lastly, the functional significance of promoter CpG methylation was validated using *in vitro* methylation of a plasmid construct with inserted promoter sequence.

In the present study, an ELA measure encompassing both childhood SES and abuse was associated with large Cohen’s *d* effect sizes in differences in 5 CpG sites within different regulatory regions of the gene. However, only one site within the first intron remained significantly associated with ELA after correction for multiple comparisons. Although prior studies have reported associations between ELA exposure and *OXTR* DNA methylation, the specific CpG sites varied across studies. Unternaehrer *et al*.^110^ reported an association between low maternal care and *OXTR* DNA methylation within the 3^rd^ exon, while Smearman *et al*.^90^ found that early abuse exposure was associated with greater methylation in two CpG sites within the *OXTR* promoter. In contrast, Needleman *et al*.^109^ reported that low childhood SES was associated with *OXTR* methylation in non-promoter regions of the genes, but not in the *OXTR* promoter. The present study investigated 3 genomic regions within *OXTR*, including an *a priori* defined region within intron 1 that has been previously associated with autism symptoms, externalizing behaviours, psychological distress, and social perception^112^, as well as the promoter region upstream of the transcription start site next to exon 1, and a distal enhancer element located within intron 3, identified using ChIP-sequencing experiment data as potentially regulatory regions of the gene. While there was no overall change in methylation in these different genomic regions, specific CpG sites within each region were differentially methylated as a function of ELA exposure among females. Results from past studies are not directly comparable with the present findings because they used different conceptualizations of ELA and they differ in the specific CpG sites assessed, with some studies relaying on commercial arrays and other studies using different a priori defined regions. This highlights the importance of using a similar set of CpG dinucleotides in future research to facilitate comparison across studies.

Among females, high ELA was related to higher mean DNA methylation in intron 1 CpG 5 and trajectories of greater childhood anxiousness were related to higher methylation of promoter CpG 7. The effect sizes of the differences in methylation between ELA and childhood anxiousness groups were equivalent to large effect sizes according to Cohen’s criteria^107^, with 8.13 and 22.22% mean methylation difference between groups. This is consistent with other studies of early life adversity, DNA methylation, and psychiatric disorders whereby mean DNA methylation differences related to phenotypic or environmental factors ranged from 1% to 5% using salivary or blood samples^23, 113–115^. Greater DNA methylation, especially in the promoter of the gene, is typically associated with lower gene expression^116^. Results of the *in vitro* luciferase experiment confirmed that greater methylation within the promoter region resulted in lower gene expression.

The oxytocinergic system is involved in the expression of social behaviors and has been related to risk for anxiety and depression. Oxytocin exerts most of its behavioural effects by binding to receptors located in the brain^35^. There is evidence that *OXTR* methylation from peripheral cells correlates with *OXTR* methylation in brain cells^76, 87^. Furthermore, a number of studies suggest that *OXTR* methylation from whole blood is significantly associated with structural and functional neural processes. *OXTR* methylation was associated with neural activation in response to ambiguous social stimuli^81^, decreased functioning coupling between the amygdala and the prefrontal cortex in response to angry or fearful faces^82^, and volumetric differences in temporal-limbic and prefrontal regions involved in social cognition^117^. In addition, *OXTR* methylation has been related to social cognition processes and styles such as emotion recognition as well as interpersonal distrust^86, 117^. *OXTR* methylation may thus lead to subtle changes in the neural networks supporting social cognition, leading to increased risk for anxiety and depression later in life. An exploratory analysis indicated that there was an indirect effect of the relationship between ELA and childhood anxiousness through *OXTR* promoter CpG 7 methylation among females. Future studies should attempt to replicate this finding in larger samples.

In this sample, *OXTR* DNA methylation was not associated with childhood trajectories of disruptiveness. This is in contrast with studies showing an association between *OXTR* DNA methylation and callous-unemotional traits among children^78^. However, other studies observed that *OXTR* methylation was associated with callous-unemotional traits only among adolescents, but not among children^77^. This suggests that our childhood measure of disruptiveness might have been less sensitive to *OXTR* DNA methylation than if it had been collected during adolescence. Alternatively, differences between our disruptiveness measure, including aspects of hyperactivity and oppositional behaviors, and the callous-unemotional trait assessments performed in other studies might also explain the discrepant results.

The association between *OXTR* DNA methylation and ELA was stronger among females than among males. These data parallel findings by Rubin *et al*.^117^ who observed sex-specific associations between *OXTR* methylation and plasma OT levels, performance at an emotion recognition task, and volumetric differences in brain regions involved in fear and social cognition. Notably, *OXTR* methylation was associated with these behavioral and physiological phenotypes among females, but not among males^117^. These data are broadly consistent with the sexually dimorphic effects of oxytocin on social behavior in different rodent species^111^ as well as with the sex differences in the effect of intra-nasal oxytocin administration in some^118–120^, but not all human studies^121^. While these results need replication, they suggest that women’s greater sensitivity to the impact of ELA on *OXTR* methylation may underlie some of the sex differences in anxiety and depression later in life^122^.

The exact mechanism through which early life experiences are translated into DNA methylation changes is not yet clear. It has been postulated that external stimuli may trigger specific signaling pathways, which then recruit DNA methylation-specific proteins to exert their actions on specific genes and gene segments^123^. For example, it is generally thought that maternal behavior triggers specific serotonin signaling pathways in the brain, followed by release of secondary messenger signals (cAMP), which recruit DNA methylation-specific enzymes that aid in targeting specific genome locations and methylating/demethylating the corresponding positions^5^. This is thought to lead to active transcription or repression of gene expression. If women are preferentially recruiting the oxytocinergic system during social interactions, this may explain the sexual dimorphism observed in this study.

ELA not only affects DNA methylation within candidate genes, but also has genome-wide and system-wide effects^124^. Given that genes do not act individually but in clusters of functional circuitries, it is likely that changes in one candidate gene will have ramifications in other genes within the same functional pathway^125^. Szyf & Bick^5^ suggest that *OXTR* DNA methylation may affect other downstream effectors within the oxytocinergic system such as the *CD38* or *OXT* genes as well as genes related to the vasopressinergic system and other genes of the same functional circuitry involved in the regulation of social behavior^126^. Future studies should assess methylation of genes within the same functional circuitry as the *OXTR* gene.

Recent studies suggest that DNA methylation of certain CpG islands is influenced by the individual’s genotype^127^. With regards to the *OXTR* gene, there is preliminary evidence that *OXTR* genotype may influence DNA methylation of specific *OXTR* CpG dinucleotides^90^. Furthermore, *OXTR* single nucleotide polymorphisms (SNPs) interacted with *OXTR* methylation to predict current anxiety and depression symptoms^90^. These *OXTR* genotype by methylation interactions predicting current psychological distress have been observed in other studies^85, 128, 129^. However, interactions of specific SNPs and CpG sites have not been consistent across these studies. Genotype information for the *OXTR* gene was not available in the current study. Future studies should assess the interaction between *OXTR* methylation and genotype in the prediction of behavioral phenotypes. Larger samples encompassing a broad representation of the different *OXTR* genotypes will be necessary to examine this issue.

This study possesses several unique strengths. The study participants were selected from a subsample of a longitudinal cohort that was recruited at the same age and from the same ethnic background, Caucasians of Western European Ancestry, minimizing age- and race-related methylation differences. A second strength is the prospective assessment for early socio-economic status during childhood rather than a retrospective assessment used in most studies. Third, our ELA assessment considered both early socioeconomic status as well as exposure to physical and sexual abuse, providing a more comprehensive assessment of early life adversity. Fourth, childhood trajectories of anxiousness and disruptiveness were teacher-rated, minimizing the risk for gene-environment correlations associated with mother- or participant-reports. Finally, the functional significance of promoter CpG methylation was confirmed using an *in vitro* luciferase experiment.

One limitation of this study is related to the timing of the DNA methylation assessment. Although *OXTR* methylation was conceptualized as the mediator of the effects of ELA, its assessment at the age of 27 followed rather than preceded the assessment of the childhood trajectories of anxiousness. This approach is defendable because ELA-related methylation has been found to be relatively stable in other studies and to be more strongly related to early life stress than current stress^17, 130, 131^. However, given that no information is available on the stability of the methylation signature of this specific gene from this specific tissue^132^, an alternative explanation is also plausible. Namely, the possibility that childhood anxiousness could also be driving the changes in methylation cannot be ruled out in this study design. Furthermore, given the correlational design of the study, no causal inference can be made from these data. Moreover, DNA methylation frequency was assessed using whole blood. It is possible that the cell type composition of our sample might have influenced the DNA methylation frequency observed^133^. Furthermore, this study did not include information about current anxiety and depression. These factors may also impact current *OXTR* methylation^83, 85^. Also, given that participants were selected for extreme exposure to ELA, it is possible that a different pattern of results may emerge among individuals with less severe exposure to ELA. Other forms of ELA such as child neglect and household dysfunction should also be assessed in other studies^97^. Furthermore, given that many CpG sites were included in the promoter construct, results of the luciferase experiment indicate that the promoter region influences gene expression, but it does not provide evidence for the role of specific CpG sites. Moreover, this study was based on relatively small number of participants. Despite large effect sizes, most of the significant associations became non-significant after adjustment for multiple comparisons due to the large number of CpG sites assessed. Furthermore, only a sex-specific correction for multiple comparisons was applied in the present study; all significant findings would become non-significant if the number of comparisons across sexes was considered for the Bonferroni correction. Given the goal to identify novel CpG dinucleotides sensitive to ELA, a relatively large number of CpG sites was evaluated in this small study, thereby increasing risk of Type I error. Replications of the present results using a priori hypotheses regarding specific CpG sites in larger samples are thus paramount.

In sum, this study provides preliminary evidence that ELA is associated with lasting changes in *OXTR* methylation and that these changes are related to phenotypic differences in internalizing symptoms among females. This suggests that the early social environment may shape the epigenetic regulation of the *OXTR* gene in a way that increases risk for anxiety and depression later in life. By identifying potential CpG sites sensitive to ELA and internalizing symptoms, these preliminary results may help uncover the molecular, physiological, and psychosocial processes underlying the protracted risk of ELA on anxiety and depression later in life among females.

## Electronic supplementary material

Supplementary Information
