# Supplementary material for: Associations among oxytocin receptor gene (OXTR) DNA methylation in adulthood, exposure to early life adversity, and childhood trajectories of anxiousness
Source: Sci Rep. 2017 Aug 7;7:7446. doi: 10.1038/s41598-017-07950-x (PMC5547144; doi:10.1038/s41598-017-07950-x)
Supplement: Supplementary file 1 — Supplementary Information [file 41598_2017_7950_MOESM1_ESM.pdf]

Associations among oxytocin receptor gene (*OXTR*) DNA methylation in adulthood, exposure to early life adversity, and childhood trajectories of anxiousness.

J.P. Gouin[*1], Q.Q.Zhou[1], L. Booij[1,2 *],M. Boivin[3,4], S.M. Côté[5,6,7], M. Hébert[8], I. Ouellet-Morin[9], M. Szyf[1,2*], R.E. Tremblay[6]*, G. Turecki[11], and F. Vitaro[6,12] (*Senior authors).

PCR Primers		
Promoter	OXTR_Promo_F1	GTTGGTTTTAGAGTTTTAATAAATGGG
	OXTR_Promo_R1	/5Biosg/ATTTTTAAATCCTAACCTTTTTTTCTAACT
	OXTR_Promo_8CG_F2	ATGGGTTTATTTTGTAGTGGTTTAA
	OXTR_Promo_8CG_R2	/5Biosg/AATAACCCCTCTCCCAACACTACTTAAAA
	OXTR_Promo_7CG_F3	ATTAGAAATGGTTTTTATTTTAAGTAGTGT
	OXTR_Promo_7CG_R3	Same as OXT_Promo_R1
Intron	OXTR_Intron_F1	GAGAGATTTTAGTTTAGTATTATATTAG
	OXTR_Intron_R1	CCCTAAACTTCCACAACACCTAC
	OXTR_Intron_F2	GTAGTTGGGTGTTAAGTAGGTAG
	OXTR_Intron_R2	/5Biosg/CAAACCCTAACATAAACACCTCC
Enhancer 1	OXTR_Enh1_F1	GTTTTGAGTTTTTGGTGATTTTTTTT
	OXTR_Enh1_R1	TTTCCCTACCCCTTAAACTTCAACA
	OXTR_Enh1_F2	GGTTTTTTGTTTTTTAGTTTTTTTATTTGT
	OXTR_Enh1_R2	/5Biosg/CACTCTAACCAAAATCTATATATATC
**Pyro Sequencing Primers**		
Promoter	Promo_SQ1	GTAGTGGTTTAAAATT
	Promo_SQ1_2	GAGAGGGAGGGAATT
	Promo_SQ2	GGTTTAGAAGTTTTTG
	Promo_SQ2_2	AGAAGTTTTTGATTT
	Promo_SQ3	ATTTTTAGTTTGATGTAG
	Promo_SQ4	GGAATATTTTTGTTTTTATT
	Promo_SQ5	GTAGTGTTGGGAGAG
	Promo_SQ6	AGTGATTTTGTTTGTTT
	Promo_SQ6_2	GTTTAAGAGT
Intron	Intron_SQ1	TTAAGTAGGGGTGGA
	Intron_SQ2	GGTGGTGGGGTGTTT
	Intron_SQ3	TTGTAAAGTGATTTT
	Intron_SQ4	TATTTGGGTTTAAAG
Enhancer 1	Enh1_SQ1	GAGTGTGTGGTTAAT
	Enh1_SQ2	TTATATTTTAAAAGTA

Supplementary Table S1. *OXTR* Targeted Regions Primers List.

CpG Reference	Genomic Position
I1	chr3:8810832
I2	chr3:8810807
I3	chr3:8810797
I4	chr3:8810774
I5	chr3:8810733
I6	chr3:8810708
P1	chr3:8811332
P2	chr3:8811348
P3	chr3:8811359
P4	chr3:8811363
P7	chr3:8811543
P8	chr3:8811601
E1	chr3:8806906
E2	chr3:8806899
E3	chr3:8806894
E4	chr3:8806887

Supplementary Table S2. Genomic coordinates for investigated CpG sites. Contains reference to exact human hg19 CpG genomic coordinates. I, P, E denote Intron 1, Promoter, Enhancer 1, respectively.

Plasmid Primers		
	Promo_S_F1<br>Promo_S_R1	TCACGGATCCAAATGGGTTTATTT<br>GTGAAAGCTTCTCAAGTCTCTCCAC
	Promo_AS_F1<br>Promo_AS_R1	TCACGGATCCTCAAGTCTCTC<br>GTGAAAGCTTTTTGCAGTGGTTTAA
Plasmid Sequencing Primers		
	Promo_SQ_F<br><br>Promoter construct sequence	GGGTTTATTTTGCAGTGGTTTAAAAC<br><br><br>AAATGGGTTTATTTTGCTCAAGTCTCTCCACAAA<br>TGGGTTTATTTTGCAGTGGTTTAAAACTGCGAG<br>AGGGAGGGAACTCGTCAATAACCCGCCCGTTTC<br>TTCCTTTCTTGGTTTAGAAGCTCTTGACTCCAGA<br>CACATAGGAGGCTTGAATAATAATGTTCTTCCC<br>CGGGTAGATAGTGATGAAGTTACAAAAGCATTT<br>AAACTGATTATTTCCCAAAAATGATGTTAATTTT<br>CAGGCTTTTCTCTCCCCCCACACCTCCAGCTTGA<br>TGTAGCGGCTTTAGCAGTGAACTCAAATAAGTC<br>TTTGCTTTGGAATACTTTTGTTCCTATTCCCGTT<br>AATGAGGAATTAGAAATGGCCTCTATTCTAAGC<br>AGTGCTGGGAGAGGCGTTATTTTTTCGGTAGTTT<br>TAGAGGAAATTCTGAAGCACAACACTGTCTTCA<br>TTTAAAGTGATTCTGCTTGTCTGCGCCCAAGAG<br>CCGTTTCTGCCTCCTTGTCGCGCTTCGGAATTAA<br>AATTCTAAATGATGCCAAAGGAAGATAAAATGT<br>TAAAGACAGCTTCTCAGCCTCCCTGCCCCTTCTC<br>TATTCTAAAATCATTTTTTGAAGTAATTTACAGT<br>GGCCAAGAATTAAGTCAGAAAAAAAGGCCAGG<br>ATTCAAAAATAAACAGGGCTCTTTAAAATGCAT<br>TAAATGTGTATTTCCTCCTGACATTTCACTTCTC<br>CGTCTTTTTATTATGTGGAGAGACTTGA G

Supplementary Table S3. Plasmid Primers List
